# Allergy to Semen: A Rare Cause of Anaphylaxis

**DOI:** 10.7759/cureus.107952

**Published:** 2026-04-29

**Authors:** Tiziana Costa, Rodianne Camilleri Agius, Mark Sammut

**Affiliations:** 1 Obstetrics and Gynaecology, Mater Dei Hospital, Msida, MLT; 2 Electrophysiology, Mater Dei Hospital, Msida, MLT

**Keywords:** allergy and anaphylaxis, gynaecology emergency, semen, semen plasma hypersensitivity, seminal fluid ige

## Abstract

A previously healthy lady in her mid-30s presented to the medical emergency with recurrent episodes of flushing, rash, wheezing, chest tightness, palpitations, dyspnoea, and loss of consciousness. After five months of intensive medical investigations, she was referred to the gynaecological team, and a detailed clinical history made it clear that these episodes always occurred after unprotected penetrative sexual intercourse. The patient was thoroughly investigated with cardiological evaluation and imaging. Eventually, a semen allergy test was performed by injecting 1 cc of semen subcutaneously in the forearm, which pushed the patient into anaphylactic shock. The patient was treated with hydrocortisone, adrenaline, and an antihistamine. The patient recovered within 24 hours. The patient was diagnosed with a semen allergy. After the diagnosis, the patient was given an EpiPen and instructed to use condoms for sexual intercourse. Barrier contraception prevented any further episodes.

## Introduction

We present the case of a female patient who has presented to the emergency room multiple times with a similar complaint. After five months of investigations, the patient was suspected of having a semen allergy since all episodes occurred after penetrative vaginal intercourse.

This case highlights the importance of a detailed medical history [1-3]. Semen allergy can present in a variety of presentations, and sometimes it may not be as easy to diagnose [3,4]. The delicate nature of the topic might deter patients from seeking medical advice. Healthcare professionals, on the other hand, may not be familiar with the diagnosis and may mistake localised symptoms for chronic vulvovaginitis [3,4].

Semen allergy can cause a psychological burden on a relationship [1], and we could see in our case that there was a sense of fear between the couple. Therefore, it is of utmost importance that a correct diagnosis be made in order to educate the patient on management and enable them to lead a normal life.

## Case presentation

A previously healthy woman in her mid-30s presented to the physicians with recurrent episodes of flushing, rash, wheezing, chest tightness, palpitations, and dyspnoea, which occurred post-coitally. She then loses consciousness. These symptoms always occur following unprotected penetrative vaginal intercourse. Each episode lasts about 30-45 minutes, followed by a headache and lethargy. No urinary incontinence, tongue-biting, or limb-jerking was present during these episodes. She denied any manifestations of local symptoms within her genital area. Her initial presentation was to the emergency department in January of 2021. She was admitted under a medical team after repeated similar presentations in May of 2021.

The patient has been with the same partner for 12 years, and they have two children together. They do not routinely use barrier contraception. The patient denied any previous atopic history and had no recollection of any close family members having allergic reactions. She is a smoker of five cigarettes daily but denies any illicit drug or alcohol abuse. She had no past medical history of note and was not on any regular treatment, and her past surgical history included two caesarean sections and one laparoscopy.

Examination between attacks was normal, including a gynaecological examination. A neurological review had also been requested after one of the attacks, and no abnormalities were noted.

Investigations

The patient was thoroughly investigated by the medical team. A CT brain showed no abnormalities, and a CT abdomen/pelvis showed ancillary findings consistent with a 4.7 cm right pericardial cyst and two hepatic haemangiomas. Urinary 5-HIAA levels were normal; therefore, carcinoid syndrome was excluded.

A transthoracic echo showed normal left ventricular dimensions and systolic function and no significant valvular heart disease. All ECGs showed normal sinus rhythm, and the loop recorder did not identify any arrhythmias.

**Figure 1:**
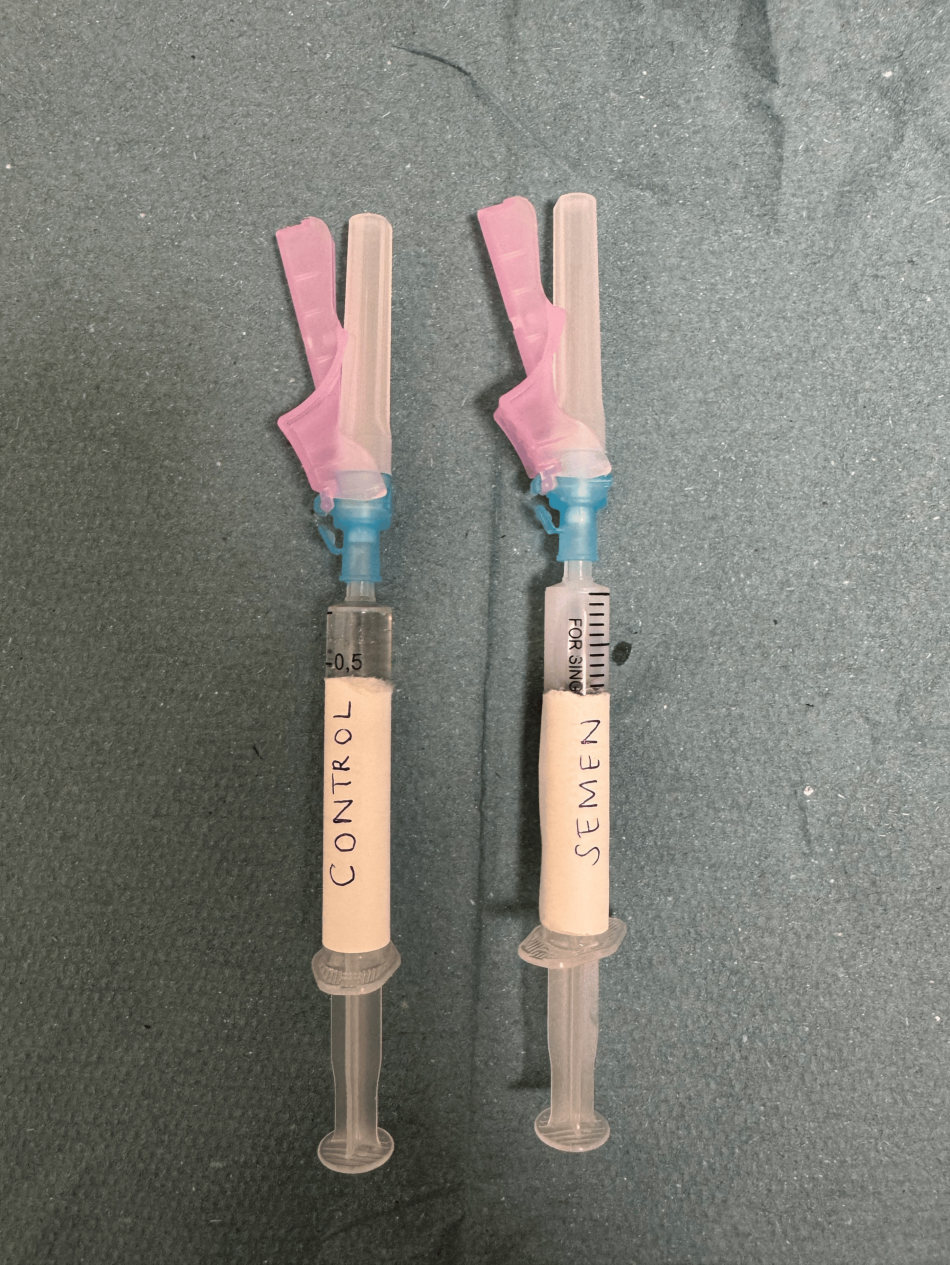
Two syringes were prepared: one as a control and one with semen

**Figure 2:**
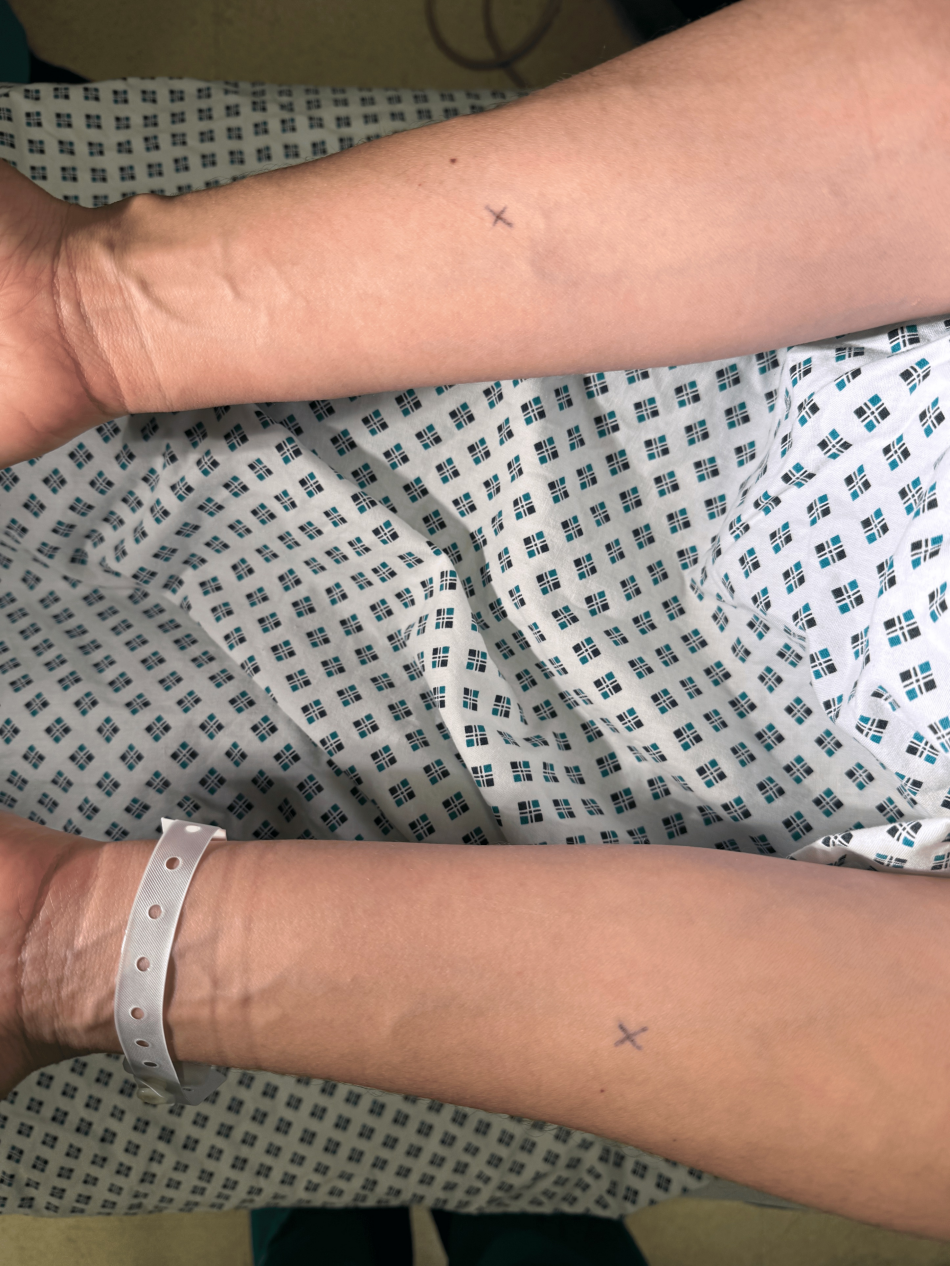
The forearms of the patient were marked before the administration of semen

**Figure 3:**
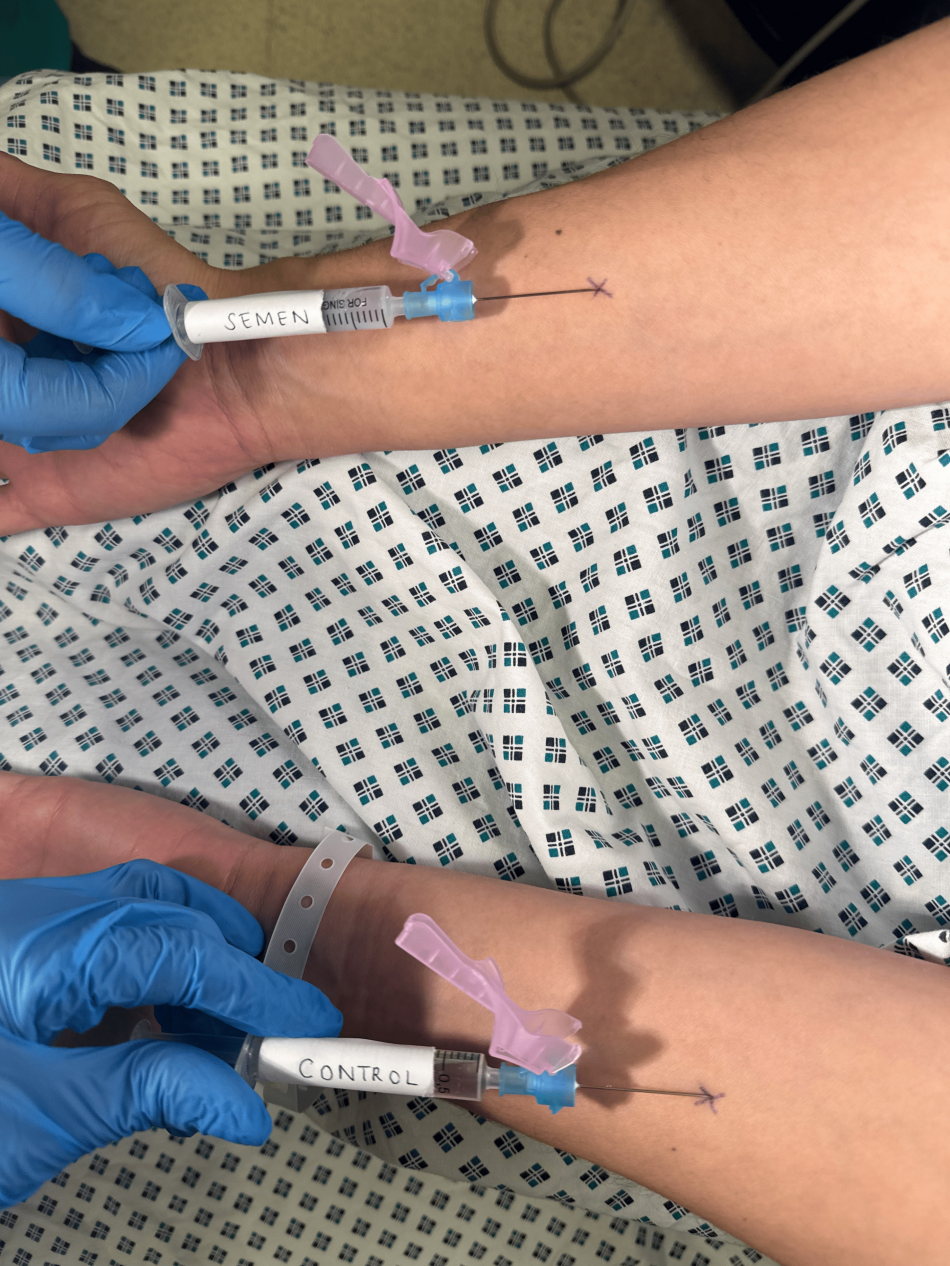
Administration of control in the left forearm and semen in the right forearm

In October 2021, the patient was referred to the gynaecology department. After obtaining consent, she was brought in for a semen allergy test. Prior to the day of testing, the partner was tested for HIV, hepatitis, and syphilis. A resuscitation trolley was prepared; 1 cc of normal saline was injected intradermally in the left forearm as a control, and 1 cc of semen was injected intradermally in the right forearm, as shown in Figures 1-3.

**Figure 4:**
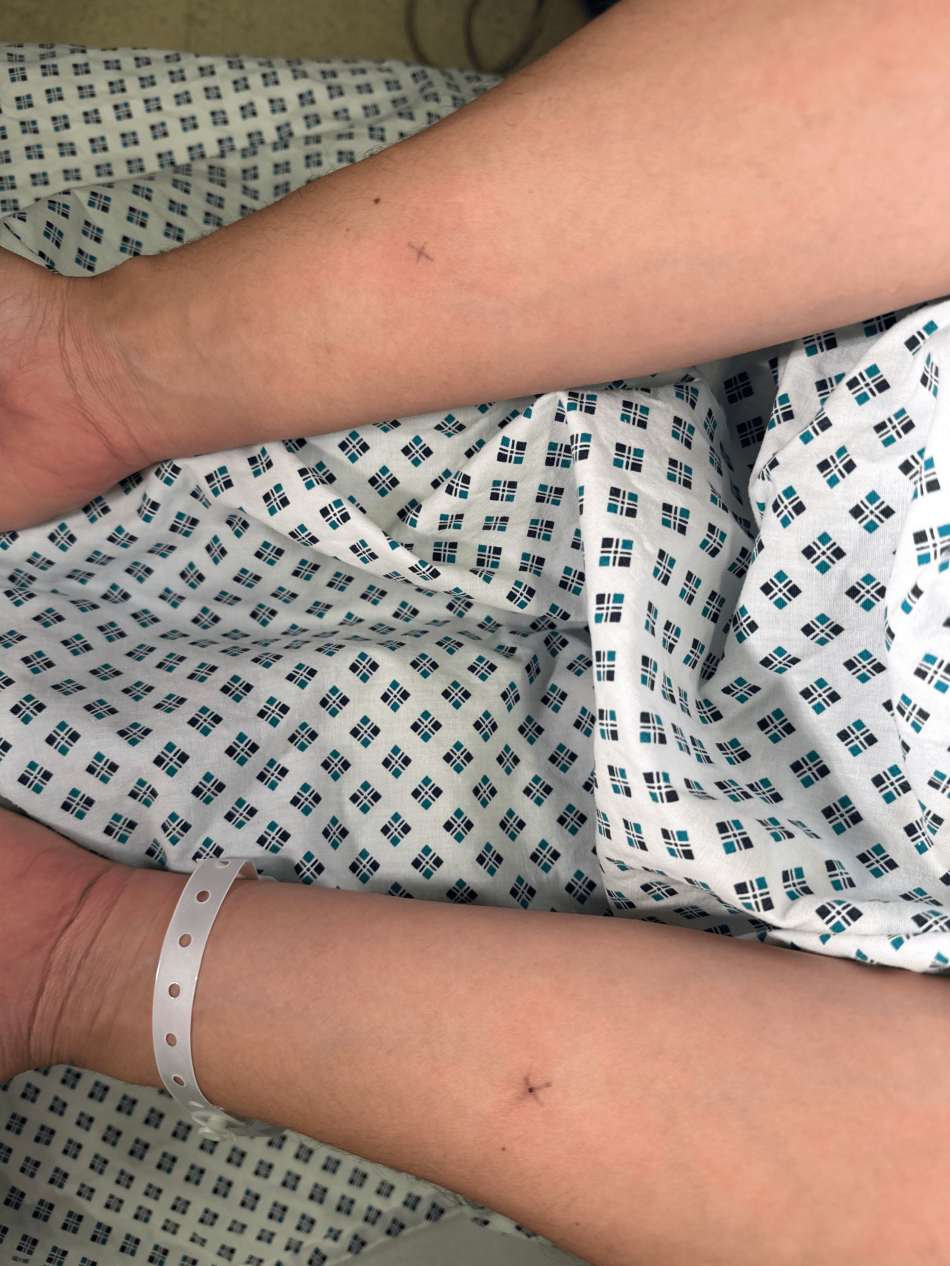
Photo of forearms after injection of control and semen

Minimal erythema at the site of administration was observed after a few seconds, as shown in Figure 4. No other skin changes occurred. The patient instantly developed dyspnoea, perioral tingling, an audible wheeze, and a maculopapular rash on the chest after administration. The patient developed a tachycardia of 104 beats per minute and hypotension, and her oxygen saturation dropped to 65% on room air. She was given hydrocortisone and promethazine intravenously and nebulised Ventolin. Intramuscular adrenaline was also given after a few minutes. The patient did not arrest; however, a CPR call was issued for resuscitation. She was then transferred to the intensive care unit for observation. She did not require invasive respiratory support and was transferred to a normal medical ward within a few hours.

The serum tryptase level was 29.2 µg/L (reference range < 11.4 µg/L) and the C1 esterase inhibitory activity was 104% (reference range 70-130%), both of which were taken on the same day of the semen allergy test. Seminal fluid IgE was checked four days after, and the level was 0.40 kU/L (reference range ≤ 35 kU/L) (Table 1). Although the IgE level is higher than the reference range, it is classified as CAP class I, meaning low concentrations of IgE antibodies were detectable.

**Table 1:** Investigations after the semen injection administration

Parameters	Patient Values	Reference Ranges
White Blood Cell Count	21.20	4.30–11.40 x 10^9^/L
Neutrophils	19.86	1.90–7.70 x 10^9^/L
Haemoglobin	13.3	12.0–15.5 g/dL
Urea	2.8	1.7–8.3 mmol/L
Creatinine	54	45–84 µmol/L
Gamma-Glutamyl Transferase	13	5–36 U/L
ALT	8	5–33 U/L
Bilirubin	7.5	0–21 µmol/L
Procalcitonin	0.514	0.02–0.046 ng/mL
CRP	1.1	0–5 mg/L
Tryptase	29.2 µg/L	<11.4 µg/L
C1 Esterase inhibitory activity	104%	70–130%

Treatment, outcome, and follow-up

The patient recovered 45-60 minutes after the administration of the injected semen. She remained lethargic and suffered a headache for the rest of the day. This was a similar response to the previous episodes that occurred after sexual intercourse. The following day, she was doing well and was discharged home. As part of the management plan, the patient was advised to strictly use condoms for every sexual encounter, and an EpiPen was prescribed. She was also referred to a respiratory physician with a special interest in allergies.

At follow-up after a few weeks, the patient confirmed that the use of condoms prevented any further reactions. This further confirmed the diagnosis.

The patient was seen again after a year, and she communicated with us that this diagnosis had a great impact on her relationship. She had ended her previous relationship and had a new one. She had unprotected sexual intercourse with her new partner, and unfortunately, she developed a similar allergic reaction again; however, it was mild. With barrier contraception, any reaction was prevented.

## Discussion

The first case of semen allergy was reported in 1958 by Specken [5]. The responsible allergens have been identified as different glycoproteins from semen, and no sperm allergens have been postulated [3]. The most common underlying mechanism is type 1 hypersensitivity involving IgE antibodies. In rare cases, hypersensitivity reactions of types 3 and 4 were documented [2,4].

There have been a few reported cases where the allergy manifested in patients with a known drug or food allergy after the male partner had ingested these specific items prior to sexual intercourse. Examples included walnut and penicillin allergies [3,6].

The initial presentation usually occurs in females aged 20 to 30 years [4]. The most common risk factor among patients is a history of atopy, such as contact dermatitis, urticaria, or asthma [4]. Most cases reported did not report the symptoms after their first sexual encounter. It is, however, the norm that after the initial allergic reaction, it tends to recur with further exposure. In most cases, symptoms occur during sexual intercourse or within 30 minutes [4].

Semen plasma hypersensitivity (SPH) is reported exclusively in females [3]. Symptoms are broadly divided into local and systemic symptoms. Local symptoms include vulval and vaginal itching, burning, and erythema. These presentations may be misdiagnosed as chronic vulvovaginitis, and in fact, SPH may be underdiagnosed [3,7]. Systemic symptoms can occur with or without localised problems and include generalised pruritus, respiratory symptoms (wheezing, dyspnoea, chest tightness, and cough), nasal congestion, sneezing, gastrointestinal symptoms (nausea and diarrhoea), angioedema, malaise, and loss of consciousness [3,4]. There were other case reports of anaphylaxis following semen exposure [3].

Diagnosis requires a good clinical history in which symptoms occur during or after exposure to ejaculate. If symptoms are avoided by condom use, the history is even more suggestive, unless there is a concurrent latex allergy [2-4]. 87% of patients experience symptoms during or within 30 minutes of sexual intercourse [4]. Once a suggestive history is elicited, other allergens and other conditions need to be excluded. A thorough gynaecological examination may help in this regard [3]. To confirm the diagnosis, a skin prick test with semen is performed. Ideally, the sample obtained from the male partner is tested for viral infections (HIV, syphilis, and hepatitis), allowed to liquefy and then centrifuged to separate the semen from the sperm. Only the semen needs to be injected, and a positive test is confirmed by a wheal of ≥3mm. To further validate the results, a negative control with saline and a positive control with histamine should be included. The male partner should also serve as a control and undergo the same test to exclude false-positive skin reactions in the female patient [1,3].

Avoidance of exposure to semen is the basis of treatment. This is achieved by condom use or abstinence from sexual intercourse [1-4]. In cases where couples find these measures inappropriate, another option is immunotherapy [1,3,4]. This involves the initial exposure of semen using the intravaginal or subcutaneous route, followed by a period of maintaining the desensitisation by regular unprotected sexual intercourse [3]. Regardless of the treatment, the patient and her partner should be provided with an EpiPen and trained on how to use it [1,3]. As caregivers, we should also consider the psychological burden on the couple after being given this diagnosis [1].

## Conclusions

This case underscores the importance of obtaining a detailed medical history when evaluating patients for SPH, as presentations may include a wide range of local and/or systemic symptoms. Raising awareness of this condition among clinicians is essential, as our local experience suggests that many physicians within the department were unfamiliar with this diagnosis. Confirmation of the condition is typically achieved through a skin prick test using semen. Once diagnosed, the cornerstone of management is the avoidance of semen exposure, which helps prevent further allergic reactions and reduces symptom recurrence.
